# Expression and Function of the Endocannabinoid Modulating Enzymes Fatty Acid Amide Hydrolase and *N*-Acylphosphatidylethanolamine-Specific Phospholipase D in Endometrial Carcinoma

**DOI:** 10.3389/fonc.2019.01363

**Published:** 2019-12-19

**Authors:** Thangesweran Ayakannu, Anthony H. Taylor, Monica Bari, Nicoletta Mastrangelo, Mauro Maccarrone, Justin C. Konje

**Affiliations:** ^1^Endocannabinoid Research Group, Reproductive Sciences Section, Department of Cancer Studies and Molecular Medicine, University of Leicester, Leicester, United Kingdom; ^2^Gynaecology Oncology Cancer Centre, Liverpool Women's NHS Foundation Trust, Liverpool Women's Hospital, Liverpool, United Kingdom; ^3^Department of Molecular and Cell Biology, University of Leicester, Leicester, United Kingdom; ^4^Department of Experimental Medicine and Surgery, Tor Vergata University of Rome, Rome, Italy; ^5^Department of Medicine, Campus Bio-Medico University of Rome, Rome, Italy; ^6^Department of Obstetrics and Gynaecology, Sidra Medicine, Doha, Qatar; ^7^Women's Wellness and Research Center, Hamad Medical Corporation, Doha, Qatar

**Keywords:** endocannabinoids, endometrial cancer, fatty acid amide hydrolase, gene expression, immunohistochemistry, *N*-acylphoshatidylethanolmine-specific phospholipase D

## Abstract

**Background:** The concentrations of three *N*-acylethanolamines (NAEs), anandamide (AEA), *N*-oleoylethanolamide (OEA), and *N*-palmitylethanolamide (PEA) are increased in the endometria of women with endometrial cancer (EC). It is widely accepted that plasma levels of these three NAEs are regulated by the actions of the rate-limiting enzymes *N*-acylphoshatidylethanolamine-specific phospholipase D (NAPE-PLD) and fatty acid amide hydrolase (FAAH), which are synthesizing and degradative, respectively. The expression and activity of these enzymes have not previously been studied in EC.

**Methods:** FAAH activity in peripheral blood lymphocytes, and transcript and protein expression for FAAH and NAPE-PLD in EC tissues were measured using enzyme, quantitative RT-PCR, and histomorphometry (of immunoreactive tissue sections), respectively. Samples were from 6 post-menopausal women with atrophic endometria (controls) and 34 women with histologically diagnosed EC. Concentrations of the three NAEs also measured in plasma and tissues were correlated with lymphocytic FAAH activity and the NAPE-PLD and FAAH transcript and protein levels.

**Results:** Peripheral lymphocyte FAAH activity was unaffected in women with EC compared to controls. The FAAH transcript expression level was significantly (*p* < 0.0001) 75% lower in EC whilst NAPE-PLD levels were not significantly (*p* = 0.798) increased. In line with the transcript data, a significant (*p* < 0.0001) tumor type-dependent 70–90% decrease in FAAH protein and significant 4- to 14-fold increase in NAPE-PLD protein (*p* < 0.0001) was observed in the malignant tissue with more advanced disease having lower FAAH and higher NAPE-PLD expression than less advanced disease. Correlation analyses also confirmed that tissue NAE concentrations were inversely related to FAAH expression and directly correlated to NAPE-PLD expression and the NAPE-PLD/FAAH ratio.

**Conclusion:** These data support our previous observation of tissue levels of AEA, OEA, and PEA and a role for NAE metabolism in the pathogenesis of EC.

## Introduction

The levels of the endocannabinoids *N*-arachidonylethanolamine (anandamide; AEA), *N*-palmityolethanolamine (PEA), and *N*-oleoylethanolamine (OEA) have been shown to be higher in endometrial cancer tissue of post-menopausal women when compared to that of controls (1). The key enzymes of the endocannabinoid system, *N*-acylphoshatidylethanolamine-specific phospholipase D (NAPE-PLD) and fatty acid amide hydrolase (FAAH), which are respectively involved in the synthesis and degradation of some of the endocannabinoids, have also been shown to be expressed and regulated in normal endometrium (2–4). Since AEA, OEA and PEA levels are higher in endometrial cancer tissues (1), we adduced that either a reduction in FAAH expression, or an increase in NAPE-PLD expression, or both, occurring simultaneously, might be responsible for the observed higher tissue levels of these endocannabinoids (collectively referred to as *N*-acylethanolamines or NAEs).

The implications of these observations and conclusions are that both the changes in NAEs and their enzymes might be involved in the pathogenesis of endometrial cancer. While the expression patterns and activities of the enzymes have been studied in various tissues (3, 5) and peripheral lymphocytes (6–9), to the best of our knowledge they have not been studied in patients with endometrial cancer (EC).

The aim of this study was therefore to investigate the changes in these endocannabinoid system components in endometrial cancer. This was achieved through measurement of the transcript levels of FAAH and NAPE-PLD in endometrial biopsies of post-menopausal women with or without EC and by correlating these with measurements of their respective protein levels. Additionally, lymphocytic FAAH activity was examined and correlated with plasma AEA, OEA and PEA concentrations to determine whether the observed higher plasma AEA and PEA concentrations in women with EC are primarily a function of endometrial tissue or lymphocytic FAAH expression.

## Materials and Methods

### Participants

Volunteers were women undergoing a hysterectomy for either endometrial carcinoma (study group) or a benign gynecological condition, such as uterine prolapse (control group), at the University Hospitals of Leicester National Health Service Trust. All provided signed informed consent to take part in the study, which had Ethical approval from the Leicestershire, Nottinghamshire, and Rutland Research Ethics Committee. Exclusion criteria were those women currently on or had been on hormonal treatment (such as hormone replacement therapy or the levonorgestrel intrauterine system) in the preceding 3 months or currently on prescription or recreational drugs. Women with chronic medical conditions or any other type of cancer were also excluded. The number of subjects and details of the types of EC and basic demographics are shown in Table 1.

**Table 1:** Details of the patients' ages and BMI within the three indicated studies.

**(A) FAAH ENZYME ACTIVITY STUDY**						
**Tissue type**		**Atrophic endometrium (6)**		**Type 1 EC (15)**		**Type 2 EC (0)**
Age (years)		60.67 ± 4.27		63.87 ± 11.11		n.d.
BMI (kg/m^2^)		26.67 ± 6.50		33.13 ± 6.64		n.d.
**(B) qRT-PCR EXPRESSION STUDY**						
**Tissue type**	**Non-malignant**	**Type 1 EC**			**Type 2 EC**	
Designation	Atrophic (6)	Grade 1 (6)	Grade 2 (6)	Grade 3 (3)	Serous (3)	Carcinosarcoma (3)
Age (years)	60.67 ± 4.27	66.17 ± 16.14	66.50 ± 10.25	72.67 ± 12.06	59.00 ± 3.46	50.00 ± 5.00
BMI (kg/m^2^)	26.67 ± 6.50	33.50 ± 8.92	32.00 ± 5.97	35.33 ± 6.11	37.67 ± 2.52	36.67 ± 6.43
**(C) ICC EXPRESSION STUDY**						
**Tissue type**	**Non-malignant**	**Type 1 EC**			**Type 2 EC**	
Designation	Atrophic (6)	Grade 1 (6)	Grade 2 (6)	Grade 3 (6)	Serous (4)	Carcinosarcoma (6)
Age (years)	60.67 ± 4.27	62.50 ± 13.90	65.17 ± 9.86	66.83 ± 7.88	70.25 ± 10.97	58.33 ± 7.42
BMI (kg/m^2^)	26.67 ± 6.50	33.00 ± 8.760	34.83 ± 5.56	31.50 ± 3.08	33.00 ± 6.83	35.50 ± 5.99

*Data in all tables are presented as the mean ± SD for both age and BMI. The numbers of samples are indicated in parentheses after the designated tissue types. Samples taken from the different groups were not significantly different to the atrophic endometrium (control); Dunnett's post-test, one-way ANOVA. EC, endometrial cancer; n.d., not determined*.

### Patient Characteristics for the FAAH Activity Assay

Only type 1 EC patients were included for the measurement of lymphocytic FAAH enzyme activity in EC, because these women had been shown to have the highest plasma AEA and PEA concentrations (1). There were no differences in the ages and BMIs of the type 1 EC patients (cases) and controls (non-malignant cases) (Table 1A). Similarly, there were no significant differences in the ages and BMIs of women with types 1 and 2 EC and the controls who provided samples for the qRT-PCR (Table 1B) or the immunohistochemistry studies (Table 1C).

### Sample Collection

#### Whole Blood

For FAAH activity measurements, venous blood (2.7 ml) was collected into pre-filled monovettes with acid citrate dextrose (ACD) anticoagulant (ACD to blood ratio 1:10). After mixing, samples were stored immediately at −80°C for later studies.

#### Endometrial Tissues

Fresh uteri obtained at hysterectomy were immediately transported on ice to the histopathology department where endometrial biopsies were obtained by a consultant gynecological histopathologist. The biopsies were divided into two; one for the measurement of transcript levels and the other for histological confirmation of the diagnosis and immunohistochemistry (IHC). Both cancer and normal tissue biopsies were washed with phosphate buffered saline (PBS) to remove excess blood and thereafter immediately stored in either RNA*later*® (Life Technologies, Paisley, UK) for 24 h before being stored at −80°C for later RNA extraction or fixed in 10% formalin and embedded in paraffin wax for histological confirmation of the diagnosis and immunohistochemistry (IHC). Sections (4 μm) of the wax embedded biopsies were cut with a microtome, placed on saline-coated slides and allowed to dry. After drying, representative sections were first subjected to hematoxylin and eosin (H&E) staining for histological confirmation of disease. This was kindly performed by a senior gynecological pathology consultant.

#### RNA Extraction and cDNA Synthesis

Endometrial tissues biopsies (100 mg) were removed from the RNA*later*® and placed into lysis/binding buffer [1 ml lysis/binding buffer solution per 100 mg of tissues (miRNA Isolation Kit)] before disruption and homogenization using a TissueRuptor (Qiagen Crawley, UK) homogenizer at medium speed for 60 s on ice. The tissue was homogenized until all visible “clumps” were dispersed and total RNA extracted using the *mir*Vana™ miRNA isolation kit (Life Technologies, Paisley, UK) according to the manufacturer's protocol. Total RNA was quantified and its purity determined using a NanoDrop 2000c spectrophotometer (Thermo Scientific, Detroit, MI, USA). At this point, the RNA concentration was standardized to 10 μg/100 μl, and genomic DNA digested by treating with a TURBO-DNAse (Life Technologies, Paisley, UK) at 37°C for 30 min. The DNAse reaction was inactivated with 10 μl of inactivation buffer and the solution centrifuged at 4°C for 90 s at 10,000 × *g*. Supernatants were subjected to first strand synthesis using the high capacity cDNA MultiScribe™ Reverse Transcriptase Kit (Life Technologies, Paisley, UK) according to the manufacturer's protocol; incubation at 25°C for 10 min, 37°C for 120 min, 85°C for 5 min, and then cooled to 4°C. The cDNA was stored at −20°C.

#### Quantitative Real-Time PCR

Quantitative Real-Time PCR experiments were performed using the validated human endogenous control assay TaqMan® Array 96-Well Plates consisting of 3 reference genes previously demonstrated to be the correct endogenous genes for atrophic and EC endometrial samples (10). Each TaqMan® gene expression assay consisted of a fluorogenic FAM™ dye–labeled MGB probe and two amplification primers (forward and reverse) provided in a pre-formulated 20× mix; 1× final concentrations were 250 nM for the probe and 900 nM for each primer. Each assay had an amplification efficiency of 100 ± 10%^1^. The TaqMan® gene expression arrays were purchased from Applied Biosystems (Life Technologies, Paisley, Scotland, UK). The TaqMan® human FAAH (Hs01038660_m1) and NAPE-PLD (Hs00419593_m1) primers and probes were similarly purchased from Applied Biosystems, as FAM/MGB dye-labeled probes. Validated Taqman® endogenous control reference genes used for normalization of the FAAH and NAPE-PLD gene expression were all VIC/TAMARA dye labeled assays purchased from Applied Biosystem and were; MRPL19 (Hs00608519_m1), PPIA (Hs99999904_m1), and IPO8 (Hs00183533_m1). RT-minus and no template controls (NTC) containing DNAse-free water instead of template mRNA were included in each run. No product was synthesized in the NTC and RT-minus confirming the absence of contamination with exogenous DNA. All reactions were performed in the final volume reaction of 20 μl consisting of 2 μl of cDNA, 8 μl of DNAse-free water and 10 μl of TaqMan® universal PCR Master Mix. The plates were run on a StepOne Plus instrument (Applied Biosystem) and the thermal cycler profile used was: 2 min at 50°C, 10 min at 95°C, and then 40 cycles of 15 s at 95°C and 1 min at 60°C. All the reactions for the reference genes were performed in triplicate (both biological and technical).

#### Identification, Localization, and Histomorphometric Analysis of NAPE-PLD and FAAH Protein Expression

Immunolocalization was performed using antibodies against FAAH (1:2,000; Alpha Diagnostics International, San Antonio, TX, USA) and NAPE-PLD (1:50; SIGMA Life Science, Stockholm, Sweden), with standard immunohistochemistry protocols as previously described (2, 3, 11–13). For negative controls, the slides were incubated with rabbit IgG diluted to the same concentrations as the primary antibodies (see Supplemental Figures 1, 2). Photomicroscopy images were taken on an Axioplan transmission microscope (Carl Zeiss Ltd., Welwyn Garden City, Hertfordshire, UK) equipped with a Sony DXC-151P analog camera (Sony Inc., Tokyo, Japan) connected to a computer, running an Axiovision image capture and processing software (Axiovision version 4.4; Carl Zeiss Ltd.). All images were captured at 200× magnification and analyzed using image analysis software (ImageScope version 10.2.2.2319; Aperio Technologies, Inc., Vista, CA) as previously described (2, 12, 14). Immunoreactivity (unbiased histoscore, H-score) was assessed semi-quantitatively by assigning scores as 0 (no staining), 100 (week staining), 200 (moderate staining), and 300 (strong staining) as determined by the software algorithm. The H-score values for the glands and stroma were determined independently and then combined to provide an overall H-score for the entire tissue.

#### Quantification of the FAAH Enzyme Activity in Peripheral Blood Lymphocytes

Lymphocytes were isolated from whole blood using the methods described by Maccarrone et al. (6), and were homogenized in ice-cold 50 mM Tris-HCl (pH 7.4) buffer and centrifuged three times at 1,000 × g for 10 min each at 4°C, with the pellet discarded after each centrifugation. FAAH activity was assayed in the supernatants by incubation with [^14^C]-AEA labeled on the ethanolamine moiety (ARC, St. Louis, MO, USA) at 37°C for 15 min in Tris-HCl buffer (pH 9.0). The reaction was stopped by the addition of ice-cold chloroform/methanol (2:1 v/v) and mixed thoroughly by vortexing and then centrifuged in order to separate the water-soluble [^14^C]-ethanolamine from residual anandamide. The radioactivity was then measured by scintillation counting as reported by Bari et al. (15).

### Statistical Analysis

Statistical analysis of the data was performed using Prism version 6.00 for windows (GraphPad Software, San Diego CA, USA, www.graphpad.com). Data that did not follow a Gaussian distribution were expressed as medians and inter-quartile range (IQR) and comparison between groups performed using Mann-Whitney U-test or one-way analysis of variance (ANOVA) followed by Kruskal-Wallis multiple comparisons analysis. Conversely, normally distributed data were analyzed by parametric one–way ANOVA with Dunnett's *ad hoc* post-analysis. In all cases a *p* < 0.05 was considered to be significant. Correlations were performed using Pearson correlation analyses.

## Results

### FAAH Enzyme Activity in Peripheral Lymphocytes Is Unaffected by Disease

**Figure 1:**
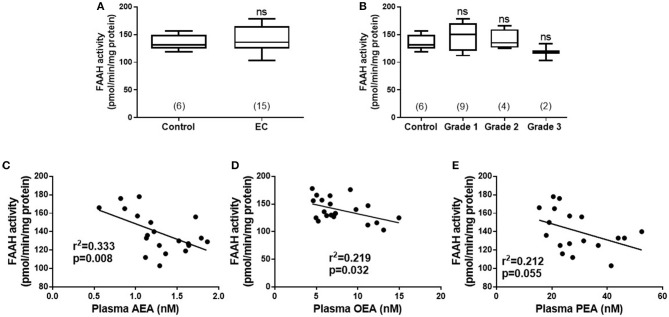
FAAH activity in peripheral blood lymphocyte membranes and relationship to plasma endocannabinoid concentrations. **(A)** Shows the FAAH activities [presented as median (IQR and range)] in lymphocyte membranes isolated from women with type 1 endometrial cancer (EC; *n* = 6) compared to that of post-menopausal women with atrophic endometria (control; *n* = 15). The EC group was subsequently divided into the different grades of type 1 EC **(B)** with (*n* = 6; control group), grade 1 (*n* = 9), grade 2 (*n* = 4), and grade 3 (*n* = 2). Spearman correlation analyses between lymphocytic FAAH activity and plasma AEA concentrations (**C**; *n* = 19), OEA concentrations (**D**; *n* = 21), and PEA concentrations (**E**; *n* = 18) are shown together with the Spearman's coefficient (*r*^2^) and *p*-value. Differences in FAAH activities **(A)** were examined by Mann-Whitney U-test and by Kruskal-Wallis one-way ANOVA with Dunn's multiple comparisons test **(B)**; ns, not significantly different. The numbers in parentheses represent the number of samples tested in each group.

Although the median (IQR) FAAH enzyme activity levels of 136.0 pmol/min/mg protein (125.0–165.0) in the lymphocytes of the EC group were higher than the 131.0 pmol/min/mg protein (125.0–149.3) in the control group; the difference was not statistically significant (*p* = 0.635) (Figure 1A). A sub-analysis of FAAH enzyme activities in the lymphocytes from the different grades of type 1 EC showed a level of 150.0 pmol/min/mg protein (120.0–170.5) in grade 1 EC, 135 pmol/min/mg protein (126.3–159.5) in grade 2 EC and 118 pmol/min/mg protein (103.0–133.0) in grade 3 EC (Figure 1B). These data were again not statistically different from that of the controls (*p* = 0.519), although the FAAH activity was increased in grade 1 and 2 EC but not in grade 3 EC (Figure 1B). There was a significant inverse correlation between plasma AEA concentrations and lymphocytic FAAH activity (*r*^2^ = 0.333; *p* = 0.008) (Figure 1C) and between plasma OEA concentrations and lymphocytic FAAH activity (*r*^2^ = 0.219; *p* = 0.032) (Figure 1D), but not between lymphocytic FAAH activity and plasma PEA concentrations (*r*^2^ = 0.212; *p* = 0.055), although this was also an inverse relationship (Figure 1E).

### Tissue FAAH Transcript Levels Are Increased and NAPE-PLD Transcript Levels Are Unaffected in EC

**Figure 2:**
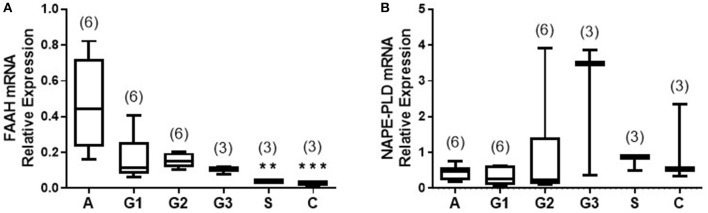
Relative expression of NAPE-PLD and FAAH transcript levels *via* qRT-PCR. The expression of NAPE-PLD transcripts **(A)** and FAAH transcripts **(B)** relative to the geometric mean of three housekeeping genes (IPO8, MRPL19, and PPIA) are shown for control (atrophic) endometria (**A**; *n* = 6), grade 1 (G1; *n* = 6), grade 2 (G2; *n* = 6), and grade 3 (G3, *n* = 3) type 1 EC tissue and serous (S, *n* = 3), and carcinosarcoma (C, *n* = 3) type 2 EC tissue. Data are presented as median (IQR). *P*-values were obtained using Kruskal-Wallis one ANOVA with Dunn's multiple comparisons test (**p = 0.0044; ***p = 0.0009). The numbers in parentheses represent the number of samples tested in each group.

The transcript levels for FAAH and NAPE-PLD, as determined by qRT-PCR relative to the geometric mean of three housekeeping genes (10), are shown in Figure 2. There was a gradual decrease in levels of FAAH transcript with tumor grade; with the highest levels of 0.445 (0.230–0.720; median and IQR) observed in atrophic endometria (controls). The levels were lower at 0.114 (0.080–0.259) for grade 1 EC, and 0.152 (0.119–1.198) for grade 2 and 0.109 (0.076–0.120) for grade 3 tumors and lower still at 0.039 (0.028–0.040) in serous cancer tissues and 0.028 (0.005–0.038) in carcinosarcoma tissue (Figure 2A). The levels in type 1 and 2 EC tissues were significantly lower (*p* < 0.01 and *p* < 0.001, respectively) than those found in control tissues.

The levels of the NAPE-PLD transcripts are shown in Figure 2B. Unlike the transcript levels for FAAH, these were not significantly different in any of the endometrial cancer types or grades when compared to control atrophic tissues (Figure 2B).

### FAAH Protein Levels Are Decreased and NAPE-PLD Protein Levels Are Increased in EC Tissues

After examining the levels of FAAH and NAPE-PLD transcripts in the different types of EC, the next step was to determine where in the tissue the proteins for these enzymes were being expressed and to confirm that the transcript changes were reflected at the protein level, by using immunohistochemistry (IHC). The details of the optimization steps taken to develop the two antibodies for FAAH and NAPE-PLD are provided in Supplemental File 1. The data indicate that both antibodies were specific for their intended antigens (data shown in Supplemental File 1).

### FAAH Protein Expression Is Decreased in EC Tissues

**Figure 3:**
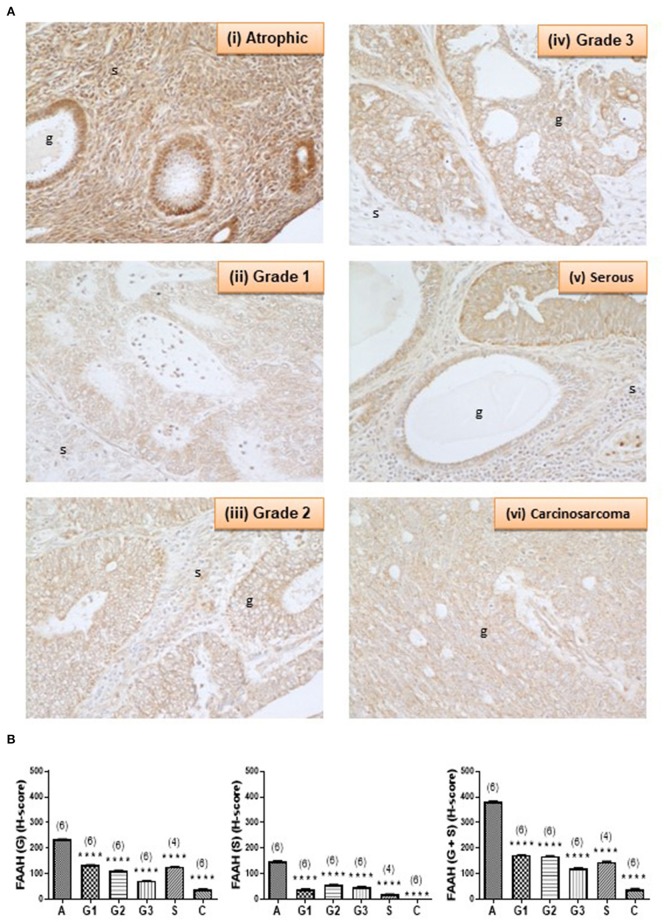
FAAH immunoreactivity decreases in endometrial cancer. **(A)** Shows representative photomicrographs of immunoreactive FAAH staining in atrophic endometria and grade 1, 2, 3 type 1 EC, and serous and carcinosarcoma type 2 EC. Note the gradual change of staining in the glandular epithelial cells (g) from less to more advanced malignancy. Also note that FAAH immunoreactivity is higher in stromal cells of the atrophic endometrium than in any of the EC tissues, and the presence of cystic glands and normal glands in the serous EC samples. **(B)** Shows data from the histomorphometric analyses of the glands alone (G, left panel) and stromal tissue alone (S, middle panel) and for the entire tissue (G + S, right panel). The H-scores for FAAH staining was statistically significantly decreased in all the EC when compared to the atrophic endometria. The results are presented as means ± SD, with one way analysis of variance with Dunnett's *ad hoc* post-test analysis used to determine the *p*-values; ****p < 0.0001 compared to atrophic; *n* = 6 in all cases except for serous EC (where *n* = 4) and numbers are shown in parentheses above each bar. Error bars are not shown when encompassed by the data.

Figure 3A shows the staining pattern of FAAH in a representative atrophic (control) endometrium and representative EC samples. Immunoreactive FAAH staining intensity was strong to very strong in both stroma and glands of atrophic endometria. In atrophic endometria, immunoreactive FAAH protein was expressed at higher levels in the glands than in the stroma with a stronger immunoreactive intensity along the basal region of the glands compared to the apical region. As might be expected from atrophic endometria, the number and size of glands were markedly reduced in the atrophic endometrium compared to all the other tissues (16).

FAAH staining in the stroma of atrophic endometria was stronger than in the stroma of other tissues and weaker than that in the glands of all tissues (Figure 3A). FAAH staining was also primarily localized to the plasma membrane of cells, with some staining demonstrated in the cytoplasm and the nucleus, whereas in other tissues, it was absent from the nuclei, but visibly attached to the nuclear membranes (see panels iii–v). FAAH staining intensity in grade 2 EC was very low compared to the atrophic group, with weaker staining in both the glands and stroma. In the glands, FAAH staining was localized more in the cytoplasm, with more intensity toward the apical region of the glands, with the nuclear envelope only slightly stained.

FAAH staining in carcinosarcoma (panel vi) showed very low intensity compared to that observed in the atrophic group, but involved both the glands and stroma. It was difficult to see the glandular structure in the carcinosarcoma, because the glands had been broken down and the epithelial cells dispersed throughout the tissue, but in those epithelial cells that were visible, FAAH staining was more cytoplasmic and localized to the apical region. Consistent with the staining in other tissues, the nuclear envelope and the nucleus had taken some staining. This was observed in both the glands and stroma. Histomorphometric analysis of these data confirmed the visual staining patterns in the glands alone, the stroma alone, or when the glands and stroma were combined (Figure 3B), with FAAH protein levels (measured as H-score) in the glands and stroma combined (right panel) being significantly lower (*P* < 0.0001) in all the various grades of type 1 [grade 1 (172.9 ± 0.76), grade 2 (169.5 ± 0.64), and grade 3 (120.3 ± 2.23)] and type 2 EC [serous (146.5 ± 1.94) and carcinosarcoma (39.18 ± 2.50)] compared to the atrophic control tissue (383.3 ± 1.10).

### NAPE-PLD Protein Expression Is Increased

**Figure 4:**
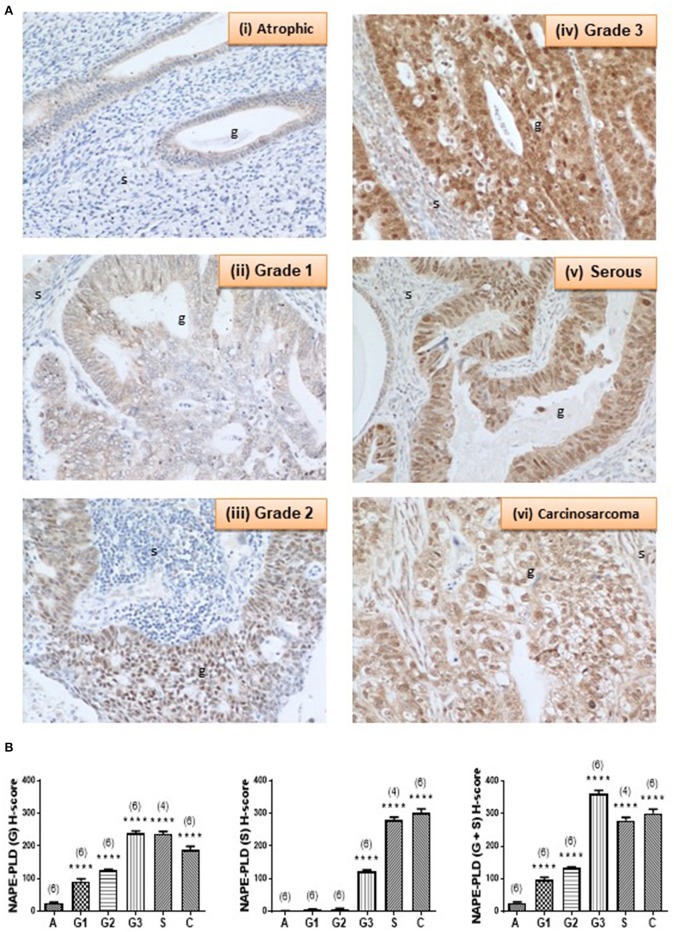
NAPE-PLD immunoreactivity increases in endometrial cancer. **(A)** Shows representative photomicrographs of immunoreactive NAPE-PLD staining in atrophic endometria and grade 1, 2, 3 type 1 EC, and serous and carcinosarcoma type 2 EC. Note the gradual change of staining in glandular epithelial cells (g) from the less to more advanced malignancy. Also note the apparent lack of staining in the stromal cells of the atrophic endometria. **(B)** Shows data from the histomorphometric analyses of the glands alone (G, left panel) and stromal tissue alone (S, middle panel) and for the entire tissue (G + S, right panel). NAPE-PLD staining was statistically significantly increased in all the EC when compared to the atrophic endometria. The results are presented as means ± SD. Statistical significance was determined using one way analysis of variance with Dunnett's *ad hoc* post-test analysis; ****p < 0.0001 compared to atrophic; *n* = 6 in all cases except for serous EC (where *n* = 4). The sample numbers are shown in parentheses above each bar.

Complementary staining patterns for the NAPE-PLD antibodies are shown in Figure 4A. In control endometria, NAPE-PLD immunoreactivity was found in the glandular epithelium with the most intense staining observed in the basal region of the glands, and away from the lumen. No significant immunoreactivity was demonstrated in the stroma. Although NAPE-PLD staining was demonstrated in the cell cytoplasm, no staining was found in the nucleus (Figure 4A, panel i). NAPE-PLD staining intensity in grade 1 EC was slightly higher when compared to that of the control tissue. The staining pattern was similar to that of atrophic tissue where it was absent from the stroma, whilst that in the glands was mainly in the cytoplasm with most nuclei not stained (panel ii). By contrast, NAPE-PLD staining intensity in grade 2 EC was more intense being greater than that of both the atrophic and grade 1 EC. Again, stroma was devoid of any staining, which was confined to the glands, where it was denser in the cytoplasm but unlike the atrophic and grade 1 EC, involved the nucleus (panel iii). NAPE-PLD staining intensity in grade 3 EC was most intense and was dramatically higher compared to the previously described groups. The staining was clearly mild in the stroma, but was very dense in the glands, where staining was found in both the cytoplasm and nucleus. In addition, some staining of NAPE-PLD was observed in the nuclei of stromal cells (panel iv).

NAPE-PLD staining in serous EC (panel v) was also higher when compared to that of the atrophic, grade 1 and grade 2 EC samples, but slightly lower than in the grade 3 EC, primarily because of decreased staining in the stroma. The staining involved both the glands and the stroma, but the glands were notably bigger compared to any other groups and stained intensely with NAPE-PLD antibodies. In the glands, the staining was denser in the cytoplasm and also present in the nucleus. Wherever the glands appeared “stretched” giving a very big cyst-like appearance; the glandular epithelium were stretched and very thin and had comparably lower NAPE-PLD immunoreactivity. In addition, NAPE-PLD was also found in the nuclei of these glandular epithelial cells with surrounding stromal cells showing both nuclear staining and light staining in the cytoplasm (panel v). NAPE-PLD staining intensity in carcinosarcoma EC was increased dramatically when compared to all the other categories, especially when compared to the atrophic (control) group. The staining involved both the glands and the stroma, with staining being denser in the epithelial cytoplasm and nucleus. In addition, NAPE-PLD staining was observed in the stromal cell nucleus (panel vi). Histomorphometric analysis of these data confirmed the visual staining patterns in the glands alone, the stroma alone, or when the glands and stroma were combined (Figure 4B) where the amount of immunoreactive NAPE-PLD in the entire tissue was higher in the EC compared to the atrophic endometria. The H-score for the types and grades of EC showed comparable data from the glandular and stromal tissues with the lowest combined H-score (26.02 ± 2.76) found in the atrophic tissues. NAPE-PLD H-scores were significantly higher in all the various grades of type 1 EC tissue (*p* < 0.0001) [grade 1 (96.48 ± 8.20), grade 2 (132.8 ± 3.10), grade 3 (360.9 ± 11.03)], as were the H-scores for the type 2 EC tissues [serous (278.9 ± 10.02) and carcinosarcoma (301.1 ± 13.10)].

### The Relationship Between NAPE-PLD and FAAH Expression

**Figure 5:**
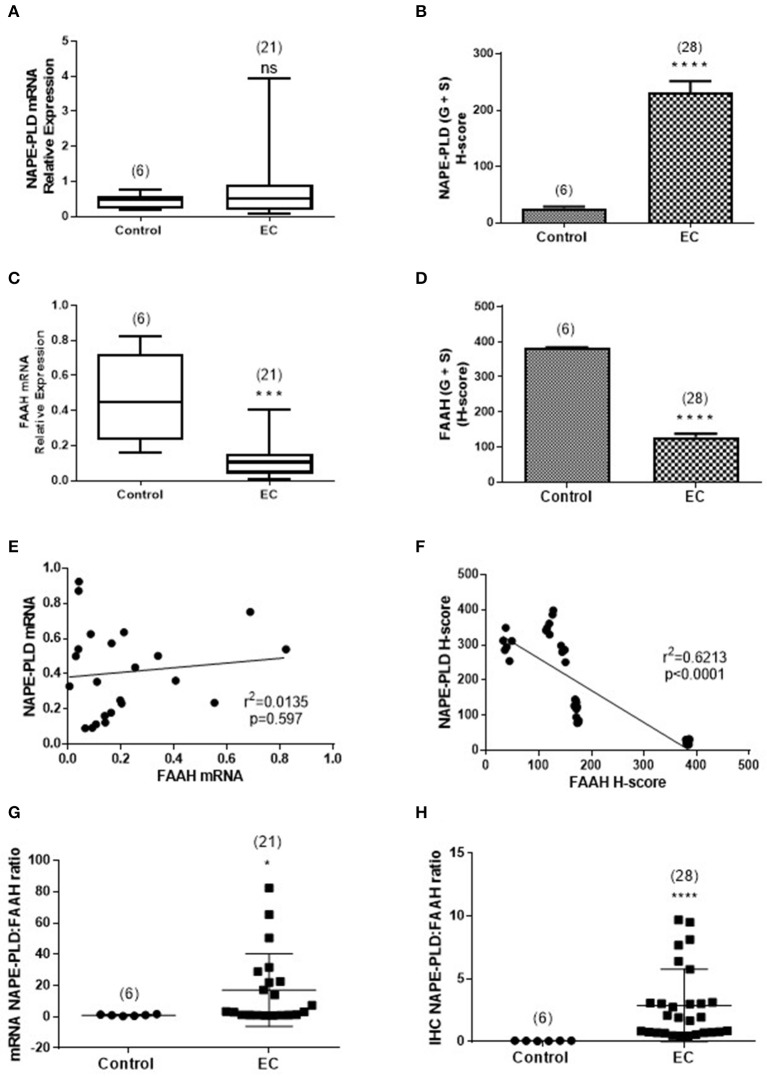
Comparison of the relative levels of transcripts for NAPE-PLD and FAAH with H-scores for the presence of protein. **(A,B)** Show the relative expressions of NAPE-PLD transcripts and protein, respectively in the atrophic tissue (atrophic control, *n* = 6) and the EC cohort (*n* = 21 for qRT-PCR and *n* = 28 for IHC). The data show similar patterns of expression with NAPE-PLD transcripts and protein increased in the EC group. Similarly, **(C,D)** show the relative expressions of FAAH transcript and protein in the same groups and show decreased transcript and protein expression in the EC group. **(E)** Shows that the expression of NAPE-PLD transcript does not correlate with FAAH transcript levels, but there is a good inverse correlation between NAPE-PLD and FAAH protein **(F)**. The qRT-PCR data **(A,C)** are presented as median (IQR) and analyzed using Mann-Whitney *U*-test, whilst the *H*-score data **(B,D)** are presented as mean ± SEM and analyzed by unpaired Student's *t*-test; ***p < 0.001; ****p < 0.0001. The number of samples analyzed is shown in parentheses above each group. Correlation was determined by Spearman correlation with the calculated coefficients and *p*-values indicated on the graphs. Error bars are not shown when encompassed by the data. The NAPE-PLD: FAAH ratios for transcript **(G)** and protein **(H)** were determined for the atrophic (control, *n* = 6) tissue and for EC (*n* = 28) tissue and the results plotted for each individual patient recruited. The longer horizontal bar indicates the mean for the data whilst the shorter horizontal bar indicates the SD for the group. The *p*-values for the transcript ratio difference (*p = 0.0208) and the protein ratio difference (****p < 0.0001) were calculated using Welch's correction for Student's two sided unpaired *t*-test for unequal variances. Error bars are not shown when encompassed by the data. The number of samples assayed is shown in parentheses above each bar.

Figure 5 shows the relationships between NAPE-PLD and FAAH transcript levels and their respective protein levels (H-score in the whole tissue) for all the combined EC samples. The data indicate that NAPE-PLD transcript (Figure 5A) and tissue proteins (Figure 5B) were increased in EC (albeit only the H-Score values were significantly different) and the FAAH transcript (Figure 5C) and protein levels (Figure 5D) were significantly decreased in EC. There was no relationship between the levels of NAPE-PLD and FAAH transcript (Figure 5E), but there was a strong inverse correlation (*r*^2^ = 0.6213; *p* < 0.0001) between the H-sores for NAPE-PLD and FAAH protein levels (Figure 5F) in the tissues. An examination of the NAPE-PLD/FAAH ratios for the transcripts and proteins showed that both transcript (Figure 5G) and protein (Figure 5H) were significantly increased in the EC tissue.

## Discussion

Both FAAH and NAPE-PLD protein expression were altered in the endometria of women with EC when compared with atrophic controls; FAAH was decreased whilst NAPE-PLD was increased. Since these metabolizing enzymes are responsible for controlling “steady state” levels of the NAEs, particularly AEA (17), they could be responsible for the increased AEA and PEA levels we previously reported for EC tissues (1). The lack of correlation between plasma NAE concentrations and lymphocytic FAAH activity in all the tumor grades (Figure 1), suggest that a concomitant loss of FAAH expression and an increase in NAPE-PLD expression in the endometrium is a possible reason for the observed higher levels of these NAEs in endometrial cancer, especially Type 1 EC. This is supported by the observation of significantly higher mRNA and protein NAPE-PLD/FAAH ratios in EC (Figure 5). Additionally, there was a good correlation between OEA levels and NAPE-PLD/FAAH expression ratios (Figure 1) indicating that although this NAE may be involved in the pathogenesis of endometrial cancer (1), its production is regulated by alterations in the activities of NAPE-PLD and FAAH at the tissue level rather than at the lymphocytic level.

It is often thought that if transcript levels are altered, then the protein levels of that gene transcript should be altered in the same manner (18); a phenomenon known as the “central dogma” of molecular biology. This, however, is not always the case (19), as has previously been demonstrated for FAAH and NAPE-PLD (20). We therefore, examined the possibility that protein expression does not exactly mirror the pattern of transcript expression and found that this indeed was the case for NAPE-PLD here, where the transcripts were not significantly higher in EC, but the protein levels were. These data suggest that only FAAH expression is likely to be involved in the modulation of tissue AEA, OEA and PEA levels in EC (1). We could have used immunoblotting techniques to complement the transcript experiments, but immunoblotting techniques do not indicate where a protein is synthesized or might act. We therefore used previously characterized quantitative IHC methods (2, 3, 11, 13) for the expression of FAAH and NAPE-PLD in EC. The findings mirrored the expected pattern of altered enzyme expression which varied with the type of tumor.

Tumor grading systems are often related to prognosis in EC with type 2 tumors considered more “aggressive” because they are independent of estrogen and furthermore are refractory to some conventional anti-hormonal treatments (21). To reduce internal experiment variations NAPE-PLD:FAAH ratios were calculated, as has been conducted before (22). This allowed the relationship between tumor grade and the expression of the enzymes to become more apparent, with a higher NAPE-PLD:FAAH ratio in higher tumor grades and thus more aggressive tumors. These data support the observed higher in plasma AEA and PEA levels that we have previously reported and suggest that they could be good biomarkers for identifying patients with EC (5).

**Figure 6:**
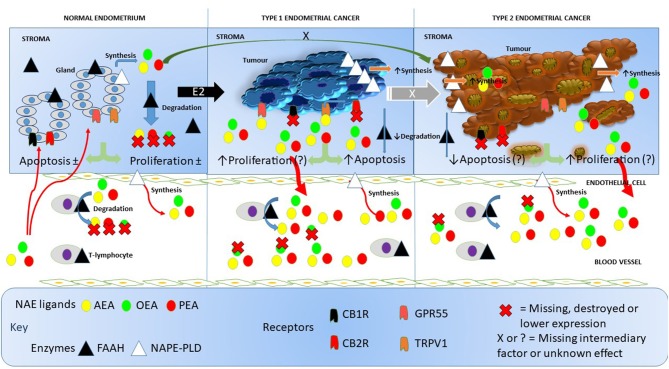
Working hypothesis on how FAAH and NAPE-PLD expression and other components of the endocannabinoid system affects NAE levels in endometrial cancer. In this figure, we depict the endocannabinoid system in normal endometrium, in type 1 EC and in type 2 EC. In the normal situation (left), endometrial tissue growth is controlled by a balance between apoptosis and cell proliferation resulting in no net gain of tissue. Relatively high levels of FAAH protein and low levels of NAPE-PLD protein keep the destruction and production of the NAEs, AEA, OEA, and PEA in the normal range within the tissue. Plasma AEA, OEA, and PEA levels are also modulated by the actions of NAPE-PLD in endothelial cells and FAAH activities in T-lymphocytes to keep plasma NAE concentrations within the normal range. The presence of excess estrogen (E2) results in malignant transformation of the endometrium into type 1 EC tissue (middle). Concomitant with that transformation is an increase in the expression of NAPE-PLD in the tissue and a loss of FAAH, cannabinoid receptor 1 (CBR1) and 2 (CB2R) (23). This allows the NAEs to bind to and activate GPR55 receptors, which may increase cellular proliferation such that it exceeds the apoptosis effect of AEA binding to the TRPV1 receptor (24). The lack of CBR1 and CBR2 receptor turnover and the increased synthesis and reduced degradation of NAEs in the tumor, results in excess release of the NAEs into the blood, where OEA is preferentially degraded by lymphocytic FAAH resulting in the “entourage effect” and the observed higher AEA and PEA concentrations seen in these patients. Less is known about the role of the endocannabinoid system in type 2 EC (right), but higher NAPE-PLD and lower FAAH, CBR1, and CBR2 expression values also results in nascent NAE production and release into the blood and similar FAAH degradation of the NAEs as found in type 1 EC. Whether the loss of CBR1 and CBR2 in type 2 EC releases the TRPV1 and GPR55 receptors to respond to the remaining NAE (or other ligands) to affect apoptosis or cell proliferation is currently unknown, as is the primary initiating factor (black X) or the factor involved in E2 desensitization (white X) that allows drug-resistant forms of type 1 EC to convert to type 2 EC. In all cases, the synthesis and degradation of the NAEs by endothelial cells and T-lymphocytes, respectively, seems to be unaffected.

To the best of our knowledge, this is the first study to quantify FAAH enzyme activity in the lymphocytes of post-menopausal women with either atrophic endometria or EC. Our data add to the generalized knowledge on the functions of NAPE-PLD and FAAH; two important regulatory enzymes considered to be “gate-keepers” in the production and degradation of NAEs, such as AEA, PEA, and OEA (17). These have allowed us to generate a working hypothesis on how the entire endocannabinoid system might be involved in EC (Figure 6) and opens up the potential to investigate targets for future research.

Whilst there have been no studies that have examined the activity of FAAH and NAPE-PLD in gynecological cancers, these have been studied in early pregnancy complications. For example, Maccarrone et al. (6), found that low FAAH activity and high AEA tissue/plasma levels were associated with miscarriage. Also interesting were the findings of decreased FAAH activity without any associated change in NAPE-PLD expression in ectopic pregnancy (13). These data suggest that in non-malignant states, the main controlling factor for plasma NAE concentrations is likely to be FAAH expression and activity. Although our data show that peripheral lymphocytic FAAH activities are however decreased in endometrial cancer compared to the atrophic controls, this reduction failed to reach statistical significance, probably due to the small number of samples studied. The lower lymphocytic FAAH activity only partly explains the higher plasma AEA concentration (1), as there was a strong statistically significant inverse correlation between plasma AEA and lymphocytic FAAH activity. The statistically significant inverse correlation with plasma OEA concentrations may be explained by the “entourage effects” of FAAH (25, 26), whereby one or more endocannabinoids are preferentially degraded, thereby potentiating the levels of AEA.

There is some evidence that FAAH and NAPE-PLD enzyme activities are directly linked to their expression (6, 27, 28). In the light of lack of enzyme activity data in the tissues we studied, the expression of the enzymes at the transcript and protein levels were used as surrogates for activity data and showed that FAAH transcript levels were significantly (*p* < 0.0001) lower by 75% in malignant tissue. Sub-analysis of the two types of EC (Figure 2A) revealed that FAAH transcript levels were significantly lower by 73.3% in type 1 EC and by 93.3% in type 2 EC when compared to controls. When FAAH transcript levels were evaluated in the various grades of cancer, they were lower in grades 1, 2, and 3 type 1 tissues and higher in type 2 serous and carcinosarcoma EC when compared to the controls (even though these data were not statistically different). Nevertheless, the data suggest that the higher plasma AEA and PEA concentrations could be controlled by the lower expression of FAAH transcript in the endometrium.

NAPE-PLD transcript levels were higher in EC but did not reach statistical significance when compared to atrophic controls. An interesting observation was that the expression of NAPE-PLD and FAAH transcripts appeared to be inversely related; whilst FAAH increased, NAPE-PLD decreased, suggesting that these two enzymes have a common regulator. This was supported by the NAPE-PLD/FAAH ratio, where a clear difference between controls and EC (Figure 5) became apparent.

The data for NAPE-PLD and FAAH protein expression followed a similar pattern to that of the transcript expression, however, the differences observed were statistically significant, with the H-scores for FAAH being lower in all EC tissues and those for NAPE-PLD all being significantly higher, regardless of grade. These data suggest that protein measurements of enzyme expression are better than transcript measurements in providing support for the suggestion that plasma AEA and PEA concentrations are possibly regulated at the tissue level, if the relationship between protein expression and enzyme activity (6, 7, 29, 30) holds true for the endometrium. What is clear is that both enzymes showed a different pattern of expression that was related to disease within the tissue.

Whilst FAAH immunoreactivity was distributed throughout the tissue, NAPE-PLD expression was primarily localized in the glandular epithelium of EC tissue with increasing intensity correlating with more advanced and aggressive forms of the disease.

In summary, the observations presented in this paper indicate that there is an apparent perturbation in the regulation of the enzymes that control local tissue and plasma concentrations of AEA and PEA (and perhaps OEA) in EC. The data also explain the observed changes in plasma NAEs of patients with EC and suggests a possible control point in the etiopathogenesis of EC.

## Conclusion

Our data show that both enzymes are expressed in EC and furthermore that they are differentially regulated with regards to the type of tumor present. The data also suggest that the main regulator of plasma AEA and PEA concentrations in post-menopausal women with endometrial cancer is unlikely to be lymphocytic FAAH activity, as has been demonstrated in other studies on endocannabinoid regulation (7–9), but is likely to be due to endometrial cancer cell FAAH and NAPE-PLD protein expression levels.

## Data Availability Statement

The datasets generated for this study are available on request to the corresponding author.

## Ethics Statement

All the volunteers gave a written informed consent to take part in the study, which was approved and conducted according within the guidelines of Leicestershire and Rutland Research Ethics Committee (reference number 06/Q2501/48) and the NHS Research Governance Framework.

## Author Contributions

The studies were conceptualized by JK and designed by TA, AT, and MM. TA performed the experiments in Leicester, whilst NM and MB performed the FAAH activity assay under the supervision of MM in Rome. All authors contributed to analyzing the data and TA wrote the first draft of the manuscript. JK was the chief investigator and the guarantor of the research. All authors contributed to the final draft of the manuscript.

### Conflict of Interest

The authors declare that the research was conducted in the absence of any commercial or financial relationships that could be construed as a potential conflict of interest.

## Footnotes

^1^Technologies, L. *Amplification Efficiency of TaqMan*^®^ *Gene Expression Assays*. Life Technologies Application Note: lifetechnologies.com (accessed October 19, 2019).
