# Supplementary material for: Expression and Function of the Endocannabinoid Modulating Enzymes Fatty Acid Amide Hydrolase and N-Acylphosphatidylethanolamine-Specific Phospholipase D in Endometrial Carcinoma
Source: Front Oncol. 2019 Dec 19;9:1363. doi: 10.3389/fonc.2019.01363 (PMC6930916; doi:10.3389/fonc.2019.01363)
Supplement: Supplementary file 1 [file Presentation_1.PPTX]

## Slide 1
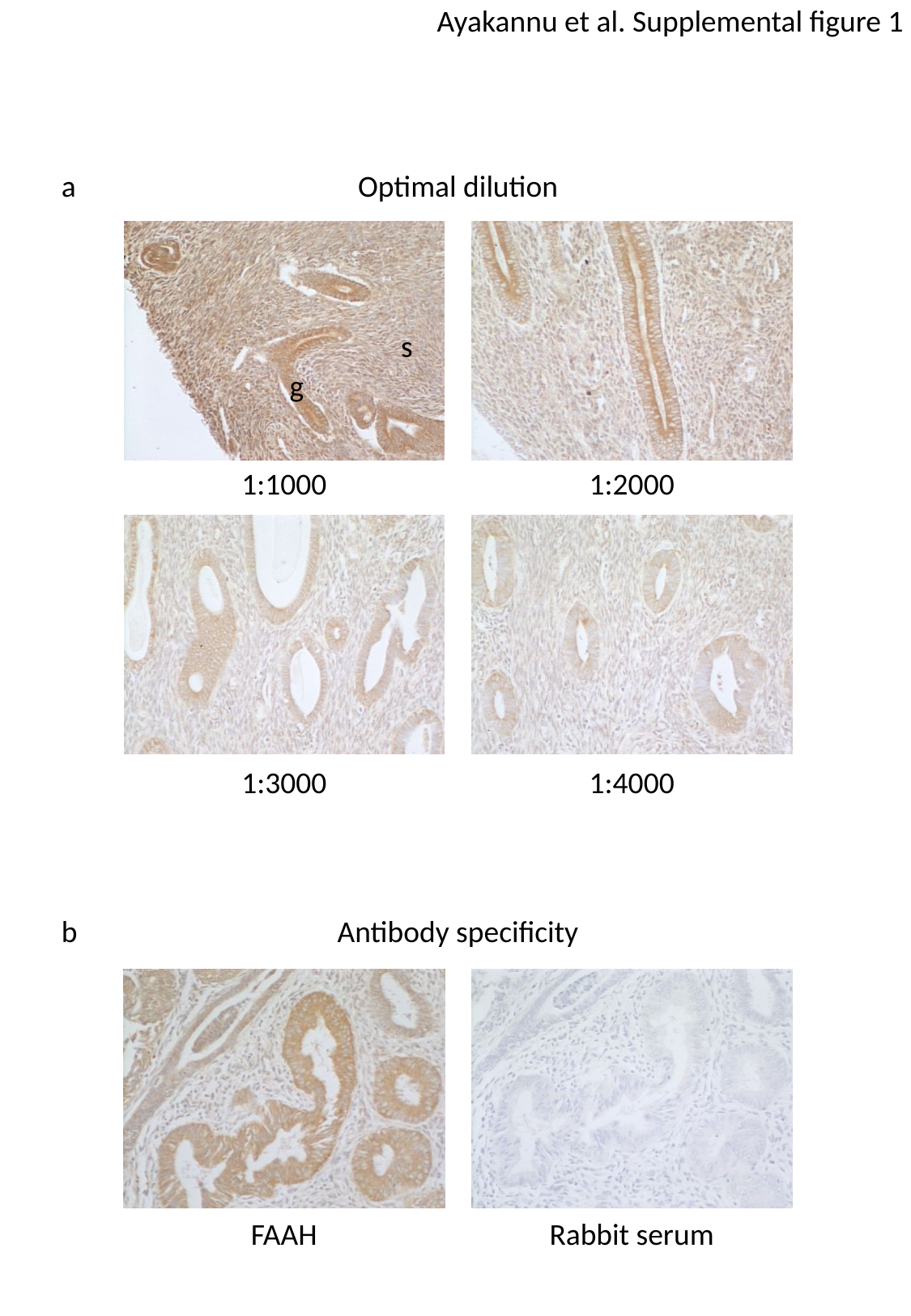

Ayakannu et al. Supplemental figure 1
a
Optimal dilution
s
g
1:1000
1:2000
1:3000
1:4000
b
Antibody specificity
FAAH
Rabbit serum

## Slide 2
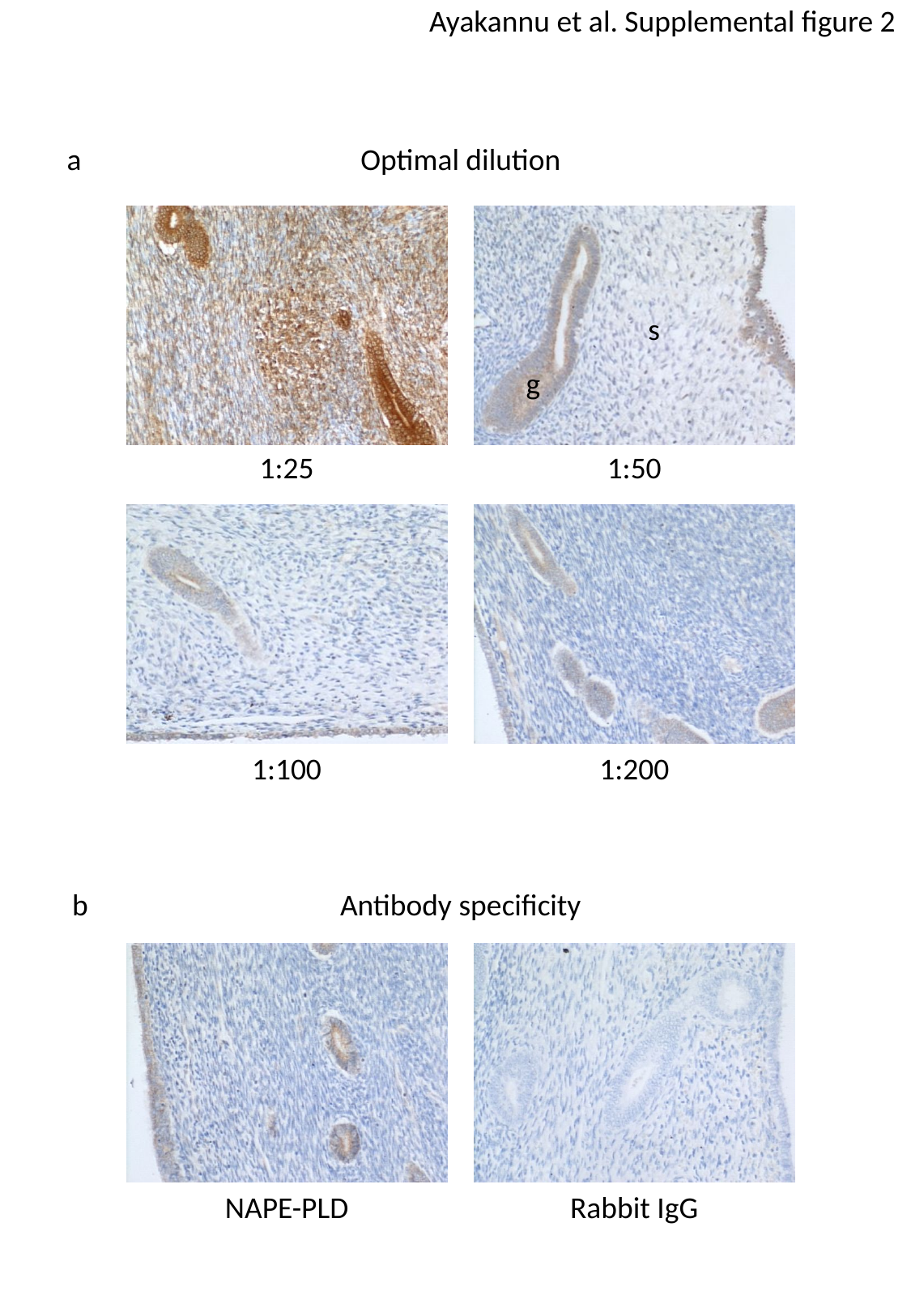

Ayakannu et al. Supplemental figure 2
a
Optimal dilution
s
g
1:25
1:50
1:100
1:200
b
Antibody specificity
NAPE-PLD
Rabbit IgG
