# Supplementary material for: Expression and Function of the Endocannabinoid Modulating Enzymes Fatty Acid Amide Hydrolase and N-Acylphosphatidylethanolamine-Specific Phospholipase D in Endometrial Carcinoma
Source: Front Oncol. 2019 Dec 19;9:1363. doi: 10.3389/fonc.2019.01363 (PMC6930916; doi:10.3389/fonc.2019.01363)
Supplement: Supplementary file 2 [file Data_Sheet_1.docx]

**Optimisation and validation of immunohistochemical methods for FAAH and NAPE-PLD protein expression.**

Immunolocalisation was performed using antibodies against human FAAH (FAAH 11-S, Alpha Diagnostics International San Antonio, TX, USA) and rabbit anti-human NAPE-PLD (catalogue number HPA024338; SIGMA Life Science. Stockholm, Sweden) with standard immunohistochemistry protocols, as previously described (1-3). For negative controls, the slides were incubated with rabbit serum (for FAAH) and rabbit IgG antibodies (Bio-Rad; for NAPE-PLD) diluted to the same concentrations as the primary antibody. Details of the procedures and image capture are presented in detail in the Methods section. All images were captured at 200 X magnification.

To determine the optimal primary antibody dilution for FAAH was obtained using 4 different dilutions (1:1000, 1:2000, 1: 3000 and 1:4000) as shown in **Supplemental Figure 1a**. The data indicated that the best dilution was 1:2000 and consequently this dilution was used for the specificity study (**Supplemental Figure 1b**). The left image shows positive staining of the tissue with the primary FAAH serum, whilst the right image shows no staining when the primary FAAH antibody was replaced non-immune rabbit serum (at the same concentration) as a control. These data indicate that the staining was specific within the endometrial samples used and similar to that previously reported (1,2).

Similar studies with rabbit anti-human NAPE-PLD antibodies required more antibody per slide. The optimal primary antibody dilution for NAPE-PLD was obtained using 1:25, 1:50, 1:100 and 1:200 dilutions of the antibody, as shown in **Supplemental Figure 2a.** The best results were obtained with the 1:50 dilution and so this dilution was used for the specificity study.

Following completing the antibody dilution optimisation study, the specificity of the optimal dilution (1:50) of the NAPE-PLD primary antibody was evaluated **Supplemental Figure 2b**. The left image shows positive staining of the tissue with the primary NAPE-PLD antibody, whilst the right image shows no staining when the primary antibody NAPE-PLD was replaced rabbit IgG (at the same concentration) as a control. These data indicate that the staining was specific within the endometrial samples used.

**Supplemental figure legends**

**Supplemental Figure 1. Optimal antibody dilution and demonstration of specificity for FAAH immunohistochemistry**

Serial sections of normal endometrium were incubated with the indicated dilutions of FAAH rabbit serum (panel a), and the antibody binding detected with 3,3’-diaminobenzidine (brown) staining. FAAH staining was noted in both the stoma (S) and the glands (G), with very strong staining observed with the 1:1000 dilution, while the 1:3000 and 1:4000 showed very light staining. The 1:2000 dilution provided the optimal staining pattern and so was used in all subsequent analyses. In panel b the specificity of the primary FAAH antibody is demonstrated with the image on the left being 1:2000 dilution of FAAH rabbit serum whilst that on the right is the non-immune rabbit serum control diluted to 1:2000 (i.e. at the same concentration as the FAAH primary antibody). Note the lack of staining in the non-immune rabbit serum control.

**Supplemental Figure 2. Optimal antibody dilution and demonstration of specificity for NAPE-PLD immunohistochemistry**

Serial sections of normal endometrium were incubated with the indicated dilutions of NAPE-PLD rabbit primary (panel a), and the antibody binding detected with 3,3’-diaminobenzidine (brown) staining. NAPE-PLD staining was noted in both the stoma (S) and the glands (G), with very strong staining observed with the 1:25 dilution, while the 1:100 and 1:200 showed very light staining. The 1:50 dilution provided the optimal staining pattern and so was used in all subsequent analyses. In panel b the specificity of the primary NAPE-PLD antibody is demonstrated with the image on the left being 1:50 dilution of NAPE-PLD rabbit antibody whilst that on the right is the non-immune rabbit immunoglobulins (IgG) diluted to the same concentration as the NAPE-PLD primary antibody). Note the lack of staining in the rabbit IgG control.

**References**

1. Taylor AH, Abbas MS, Habiba MA, Konje JC. Histomorphometric evaluation of cannabinoid receptor and anandamide modulating enzyme expression in the human endometrium through the menstrual cycle. Histochemistry and Cell Biology. 2010;133:557-65.
2. Gebeh AK, Willets JM, Marczylo EL, Taylor AH, Konje JC. Ectopic pregnancy is associated with high anandamide levels and aberrant expression of FAAH and CB1 in fallopian tubes. Journal of Clinical Endocrinology and Metabolism. 2012;97:2827-35.
3. El-Talatini, MR, Taylor AH, Elson JC, Brown L, Davidson A, Konje JC. Localisation and function of the endocannabinoid system in the human ovary. PLoS One. 2009; 4(2):e4579.
